# The potential role of inhaled nitric oxide for postexposure chemoprophylaxis of COVID-19

**DOI:** 10.1186/s43141-021-00249-5

**Published:** 2021-10-22

**Authors:** Antoine AbdelMassih, Rafeef Hozaien, Meryam El Shershaby, Aya Kamel, Habiba-Allah Ismail, Mariem Arsanyous, Nadine El-Husseiny, Noha Khalil, Youstina Naeem, Raghda Fouda

**Affiliations:** 1 Pediatric Cardiology unit, Pediatrics’ Department, Faculty of Medicine, Cairo University, Cairo, Egypt; 2 Pediatric Cardio-Oncology Department, Children Cancer Hospital of Egypt, Cairo, 57357 Egypt; 3 Research Accessibility Team, Student and Internship research program Faculty of Medicine, Cairo University, Cairo, Egypt; 4 Faculty of Dentistry, Cairo University, Cairo, Egypt; 5 Pixagon Graphic Design Agency, Cairo, Egypt; 6 Clinical and Chemical Pathology Department, Faculty of Medicine, Cairo University, Cairo, Egypt

## Abstract

**Background:**

Several vaccines have been fast-tracked in an attempt to decrease the morbidity and mortality of COVID-19. However, post-exposure prophylaxis has been overlooked in battling COVID-19.

**Main text:**

Inhaled nitric oxide is a potential tool in post-exposure prophylaxis of COVID-19. It decreases cytosolic calcium levels, which impairs the action of Furin. SARS-CoV-2 uses Furin to replicate in the respiratory tract.

**Short conclusion:**

Inhaled nitric oxide could decrease the viral load in the upper respiratory tract, abort clinically symptomatic infection, and prevent subsequent complications. Nitric oxide might be a tool for post-exposure chemoprophylaxis in at-risk groups, especially medical personnel.

## Background

SaNOtize (Canada, Vancouver-based/NCT04443868 biotech firm) recently created a self-administered nitric oxide nasal spray (NONS) that could potentially reduce coronavirus disease 2019 (COVID-19) viral load in infected patients. After completing early-stage clinical trials in Canada and the United Kingdom (UK), SaNOtize, Ashford and St. Peter’s Hospitals, the National Hospital System (NHS) foundation, and a few pathology services in the UK announced the results of phase II trials. The results indicate that NONS can be a powerful and safe antiviral treatment. It could prevent COVID-19 transmission, shorten its duration, and reduce the severity of its symptoms.

Some reports have discussed the use of nitric oxide against COVID-19. Lotz et al., for example, highlighted its potential to improve acute respiratory distress syndrome in COVID-19.

However, SaNOtize’s clinical trial results suggest that it has a much earlier antiviral role against COVID-19. We will discuss the exact mechanism behind this in this report [1].

## Main text

### Protease is critical to determine the viral load of COVID-19

Furin is a member of the PCSK (pro-protein convertase subtilizing/Kexin) family. Furin is a type 1 membrane-bound protease utilized by multiple pathogens including human immune deficiency virus (HIV), Ebola virus, Marburg virus, severe acute respiratory syndrome coronavirus 2 (SARS-CoV-2), and even some bacterial toxins. Pathogenicity can increase several folds once they react with Furin and other pro-protein convertases. After Furin is cleaved, latent precursor proteins are activated. Hence, Furin-dependent infections may respond to therapeutics targeting host cell Furin [2].

The spike protein of SARS-CoV-2 is the cleavage site of Furin. It plays an essential role in the pathogenesis, host range, and infectivity of the virus. Furin requires a polybasic instead of a monobasic cleavage site. Hence, cleavage occurs at the junction of the two polybasic, spike protein subunits (S1 and S2). High virulence and low virulence influenza strains have different pathogenicities and are an excellent example of the relationship between viral pathogenicity and cleavage sites [3].

### Nitric oxide is an inhibitor of viral proteases and subsequently of viral replication

Previous studies have noted that the antiviral role of nitric oxide is due to its inhibition of viral protease activity. It also prohibits viral replication. In a study, several viruses demonstrated the mechanism behind this phenomenon. These included coxsackievirus, picornaviruses, hantavirus, herpesvirus, rhinovirus, Japanese encephalitis, vaccinia, retrovirus, and many more (Table 1 exposes the clinical and laboratory trials which used NO as an antiviral agent) [4–24].

**Table 1:** Review of in vivo and in vitro studies of the antiviral effect of nitric oxide

Reference number in text	Virus	Type of nitric oxide therapy	Study model	Main outcome
[4, 5]	SARS-CoV	NO donor, SNAP	In vitro	Inhibited SARS CoV replication cycle in a concentration-dependent manner (1)
		NO donors, SNAP and SNP	In vitro	SNAP and SNP inhibited the SARS CoV viral cytopathic effect (2)
[7, 8]	SARS-CoV-2	inhaled NO	Multicenter randomized controlled trial	Ongoing, antiviral effect of high concentrations of inhaled NO administered during early phases of COVID-19 on spontaneous breathing patients, effect on disease progression (3)
				Ongoing, testing inhaled Nitric Oxide in mechanically ventilated patients with severe acute respiratory syndrome in COVID-19 (SARS-CoV-2) (4)
			Single-center, randomized (1:1) controlled, parallel-arm clinical trial	Ongoing, prophylactic therapy to reduce the instance of COVID-19 disease in healthcare workers (4)
		SNAP	In vitro	SNAP delayed or completely prevented the development of viral cytopathic effect (5)
[9]	Coxsackievirus	NO donors SNAP	In vitro Murine model	NO inhibits CVB3 replication by inhibiting protease activity and interrupting the viral life cycle (6)
		iNO, SNAP		NO inhibits CVB3 replication in part by inhibiting viral RNA and protein synthesis (7)
		NO donors SNAP, PFC, GTN, ISDN)		In vitro NO showed inhibition of the 2A proteinase activity CVB3-infected mice showed significantly reduced signs of myocarditis after treatment with GTN or ISDN (8)
[10, 11]	Influenza	Gaseous nitric oxide (gNO)	In vitro	Viral NA inhibition by gNO was shown and may be responsible for this antiviral effect (9)
		SNAP		inhibition of influenza virus viral RNA synthesis (10)
[12]	Japanese encephalitis virus (JEV)	SNAP	In vitro	NO was found to profoundly inhibit viral RNA synthesis, viral protein accumulation, and virus release from infected cells (11)
		MDF to produce NO (inducible NO)	In vitro and murine model	MDF stimulated macrophages inhibited virus replication with high levels of NO production. MDF treatment increased the survival rate of JEV infected mice (12)
[22]	Rhinovirus	Nitric oxide donor (NONOate)	In vitro	(NONOate) inhibited both rhinovirus replication and cytokine production in a dose-dependent fashion without reducing levels of cytokine mRNA (13)
[14]	Reovirus	iNO	In vitro	Cytostatic effects antiviral effects e.g. reduction in DNA synthesis, protein synthesis & mitochondrial metabolism (14)
[15]	Dengue virus (DENV)	SNAP	In vitro	NO showed an inhibitory effect on viral RNA synthesis. The activity of the viral replicase was suppressed significantly (15)
[16]	Herpes simplex virus type 1 (HSV 1)			Nitric oxide had inhibitory effects on HSV1 protein and DNA synthesis as well as on cell replication (16)
[17]	Porcine circovirus type 2 (PCV2)	NO generated from (GSNO)	In vivo, in vitro (Murine model)	NO strongly inhibited PCV2 replication in vitro. NO reduced the progression of PCV2 infection in mice (17)
[18]	Crimean Congo hemorrhagic fever virus (CCHFV)	SNAP	In vitro	NO reduced virion progeny yield with a reduction in expression of viral proteins; the nucleocapsid protein and the glycoprotein, and vRNA (18)
[19]	Respiratory Syncytial Virus (RSV)	iNO , SNAP	In vitro	NO has significant direct antiviral activity against RSV, which is more potent with continuous, endogenous NO production than exogenous NO (19)
[13]	Human papillomaviruses (HPVs)	NVN1000, Topical NO-releasing polymer	In vitro	NO abrogated HPV-18 progeny virus production. Reduced HPV-18 E6 and E7 oncoproteins. Impaired S-phase progression and induced DNA damage in infected cultures (20)
[20]	Vesicular stomatitis virus (VSV)	iNO, SNAP	In vitro	anti-VSV effects of NO in form of significant inhibition of productive VSV infection (21)
[21]	Molluscum contagiosum	Topical acidified nitrite, nitric oxide liberating cream)	A double-blind, group-sequential clinical trial	75% cure rate in the active treatment group NO is an effective therapy with a 75% cure rate in the treatment group compared to 21% in the control group (22)
		topical SB206 (NO releasing topical gel)	multicenter, randomized, double-blind, vehicle-controlled clinical trial	SB206 is an effective therapy with (SB206 12% / once daily) provided the best balance between MC lesion clearance and tolerability (22)
[6]	Hantavirus	iNO, SNAP	In vitro, murine model	NO strongly inhibited hantavirus replication in vitro. The viral titers in iNOS^–/–^ mice were higher compared to the controls, suggesting that NO inhibits hantavirus replication in vivo (23)

Abbreviations: *NO* nirtic oxide, *SNAP* S-nitroso-N-acetylpenicillamine, *GTN* glyceryl trinitrate, *ISDN* isosorbide dinitrate, PFC: 4-phenyl-3-furoxancarbonitrile, *iNO* inducible NO, *CVB3* coxsackievirus B3, *gNO* gaseous nitric oxide, *NA* neuraminidase, *JEV* Japanese encephalitis virus, *MDF* macrophage-derived neutrophil chemotactic factor, *NONOate* 3-(2-hydroxy-2-nitroso-1-propylhydrazino)-1-propanamine, *HSV1* herpes simplex virus type 1, *DENV* dengue virus, *PCV2* porcine circovirus type 2, *GSNO* S-nitrosoglutathione, *CCHFV* Crimean Congo hemorrhagic fever virus, *RSV* respiratory syncytial virus, *VSV* vesicular stomatitis virus

### Nitric oxide inhibits viral protease activity by decreasing intracellular cations

Furin is a cellular protease enzyme expressed from the FURIN gene in humans. Furin shows an intriguing interplay between intracellular ions, especially cations. Potassium ions are the most common intracellular ions in our bodies, followed by magnesium—which can activate Furin directly. Molloy et al. noted that the intracellular calcium level noticeably influences the activity of Furin. Thus, Furin is a calcium-dependent enzyme [25].

Yamada and colleagues further supported the relationship between Furin and calcium levels. Inhibiting Furin prevented further neuronal damage caused by calcium influx after hypoxic injury [26]. Hence, impeding calcium channels can be a promising approach against Furin-activated organisms. Additionally, Li et al. stated in 2019 that calcium channel blockers (CCB) decrease the intensity of fever spikes and the occurrence of thrombocytopenia syndrome, categorized by manifestations of tick-borne hemorrhagic fever [27].

Nitric oxide encourages calcium efflux from cells, leading to decreased intracellular calcium levels. Van Hove et al. demonstrated this and proved that nitric oxide stimulates smooth muscle cells (SMCs) to relax directly or indirectly by decreasing the elevated calcium level [28]. As such, nitric oxide could inhibit Furin’s action by decreasing cytosolic levels of calcium.

### Inhaled nitric oxide as post-exposure prophylaxis

Argyropoulos et al. concluded that a diagnostic viral load has no prognostic value [29]. While in a more recent report, Silva et al. found the saliva viral loads to be significantly higher in patients with chronic respiratory conditions, cardiovascular conditions, kidney disease, and diseases that compromise the immune system [30]. Patients with four or more risk factors had much higher saliva viral loads than patients with fewer risk factors, as did male patients. However, there was no relation between nose and throat viral loads and risk factors. Saliva viral loads were also higher in patients with worse clinical outcomes. As such, early interruption of viral replication in the upper respiratory tract might abort the development of significant symptoms and complications. This rationale might have led to the current inclusion criteria of SaNOtize’s ongoing clinical trial, which involves administration of the intranasal medication within 48 h of a diagnosis. SaNOtize could potentially be administered to medical personnel as post-exposure chemoprophylaxis.

## Conclusion

Early reports of the role of nitric oxide in the treatment of COVID-19 suggested its use for the treatment of established acute respiratory distress syndrome. However, nitric oxide seems to have a much earlier and more efficient prophylactic role. It inhibits Furin, a protease needed for canonical viral replication of SARS-CoV-2, by decreasing cytosolic calcium levels. This action can prevent the exponential increase of viral load in the upper respiratory tract leading to the abortion of clinically symptomatic infection and subsequent complications. Nitric oxide could be a tool for post-exposure chemoprophylaxis in the at-risk groups, especially medical personnel.

**Fig. 1:**
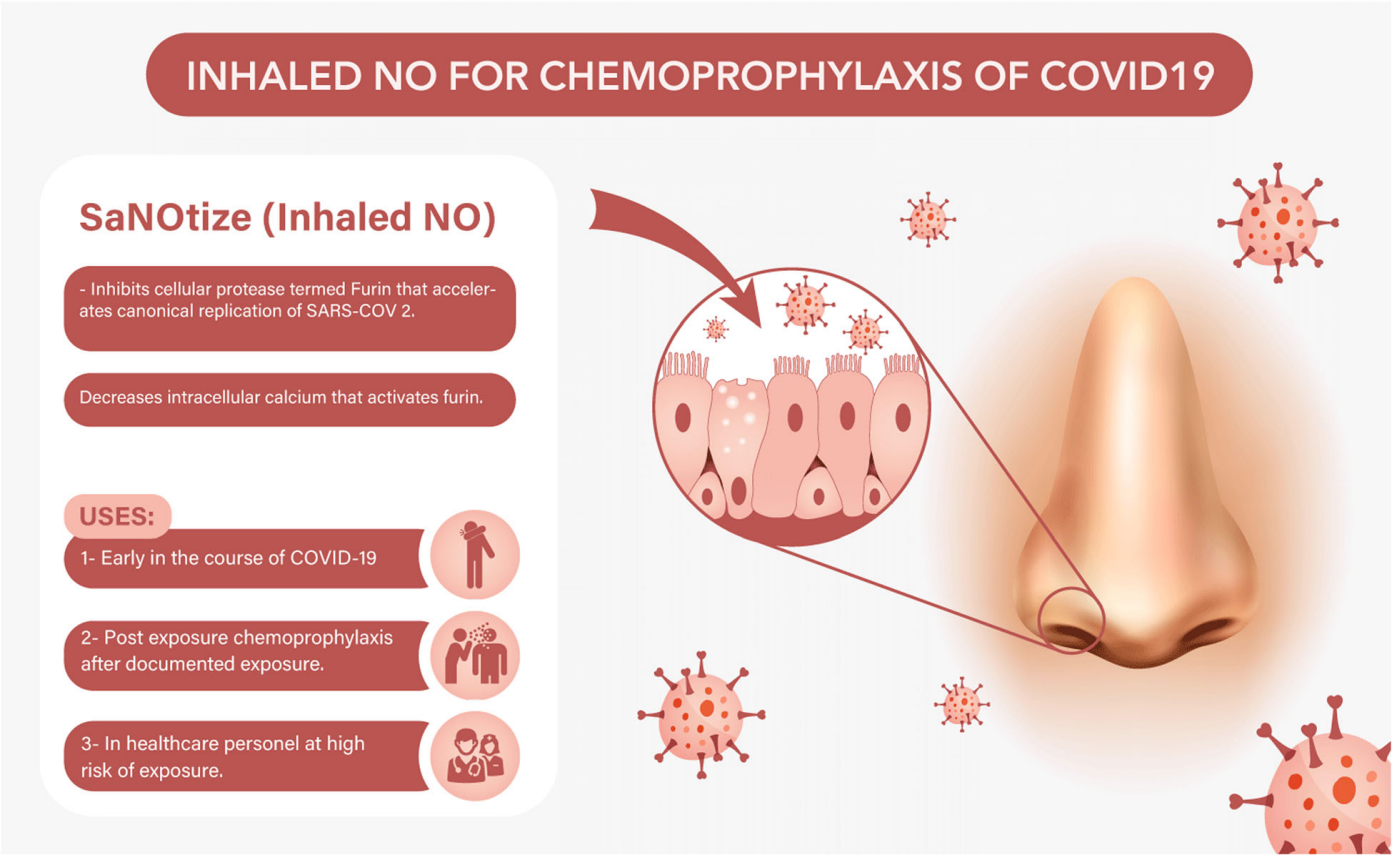
Inhaled NO for chemoprophylaxis of COVID-19. Abbreviations: COVID-19, coronavirus 2019; NO, nitric oxide; SARS-CoV-2, severe acute respiratory syndrome coronavirus 2

Figure 1 summarizes the antiviral effect of nitric oxide and its possible uses in the context of COVID-19.

## Data Availability

Not applicable

## Abbreviations

CCB: Calcium channel blocker
COVID-19: Coronavirus 2019
NHS: National Hospital System
NO: Nitric oxide
NONS: Nitric oxide nasal spray
SARS-CoV-2: Severe acute respiratory syndrome coronavirus 2
UK: United Kingdom
